# Draft Genome Sequences of Two Basal Members of the *Anaerolineae* Class of *Chloroflexi* from a Sulfidic Hot Spring

**DOI:** 10.1128/genomeA.00570-18

**Published:** 2018-06-21

**Authors:** L. M. Ward, S. E. McGlynn, W. W. Fischer

**Affiliations:** a Department of Earth and Planetary Sciences, Harvard University, Cambridge, Massachusetts, USA; b Earth-Life Science Institute, Tokyo Institute of Technology, Meguro, Tokyo, Japan; c Division of Geological and Planetary Sciences, California Institute of Technology, Pasadena, California, USA

## Abstract

Here, we describe the first genome sequences of the *Anaerolineae* from a sulfidic environment, expanding the environmental distribution of sequenced *Anaerolineae*. These genomes represent basal *Anaerolineae* lineages, branching soon after the divergence of the sister class “Candidatus Thermofonsia,” expanding our understanding of the metabolic evolution of this group.

## GENOME ANNOUNCEMENT

Although members of the *Anaerolineae* class of the *Chloroflexi* phylum appear in diverse environmental 16S sequence data sets (e.g., carbonate tidal flats [1] and iron-rich hot springs [2]), the environmental distribution of previously sequenced *Anaerolineae* is primarily limited to groundwater and wastewater systems (e.g., references 3 and 4). Here, we present the first genomes of *Anaerolineae* from sulfidic environments, Nak19 and Nak57, expanding the genetic and environmental distribution of sequenced representatives of this clade.

The metagenome-assembled genomes (MAGs) were recovered from metagenomic sequencing of microbial communities from a sulfidic hot spring in Japan, as described previously (5, 6). In brief, samples were collected from microbial mats at Nakabusa Onsen in Nagano Prefecture, Japan, and DNA was extracted and sequenced via Illumina HiSeq. Sequences from four samples were coassembled with MegaHit version 1.1.2 (7) and genome bins constructed based on differential coverage using Metabat (8). Genome bins were assessed for completeness and contamination using CheckM (9) and uploaded to RAST for overall characterization (10).

MAG Nak19 totals 3.45 Mb recovered as 158 contigs, encoding 3,163 coding sequences and 44 tRNAs. The Nak19 genome has 51.2% GC content and was estimated by CheckM to be 90% complete, with 6.09% contamination. Nak57 is 3.77 Mb over 159 contigs, encoding 45 tRNAs and 3,391 coding sequences. Its GC content is 51.2%. CheckM estimates Nak57 to be 95.45% complete, with 4.85% contamination.

Neither Nak19 nor Nak57 recovered a 16S gene, but phylogenies based on RpoB and concatenated ribosomal proteins robustly place these MAGs as basal *Anaerolineae*, with Nak19 being the basal-most *Anaerolineae* and Nak57 being more closely related to cultured *Anaerolineae*, such as Thermanaerothrix daxensis (11) and Ornatilinea apprima (12).

Nak19 and Nak57 both encode aerobic respiration via an A-family heme-copper oxidoreductase (HCO) complex, a *bd* oxidase, and a *bc* complex. Nak57 also encodes an alternative complex III (ACIII), while Nak19 encodes NirS for nitrite reduction. Phylogenetic analysis of the respiration genes from Nak57 show gene relationships congruent with organismal relationships, supporting the vertical inheritance of aerobic respiration in the *Anaerolineae* following acquisition at the base of the class, consistent with trends in other *Chloroflexi* classes (6, 13). ACIII was not recovered in Nak19, but this may be a false-negative result due to incompleteness of the MAG; however, as the basal-most *Anaerolineae* member, it may also be that ACIII had not yet been acquired by the *Anaerolineae* when this clade diverged. ACIII may therefore have been acquired on the branch leading to Nak57 and the other *Anaerolineae*. The *Anaerolineae* have typically been described as obligately anaerobic fermenters (e.g., references 14 and 15), but genes for aerobic respiration appear to be widespread in this group (3, 4, 11). It remains uncertain whether these genes are used for aerobic respiration or only for O_2_ detoxification in the *Anaerolineae* whose genomes are described here and elsewhere.

Consistent with other reports from sequenced *Chloroflexi*, Nak19 and Nak57 do not appear to encode outer membrane proteins, supporting interpretations of a monoderm membrane architecture as distinct from that of the diderm sister phylum *Armatimonadetes* (6, 16, 17).

### Accession number(s).

These whole-genome shotgun projects were deposited in DDBJ/EMBL/GenBank under the accession numbers QEXX00000000 (Nak19) and QEXW00000000 (Nak57).
